# Adverse Events in Treating Smear-Positive Tuberculosis Patients in China

**DOI:** 10.3390/ijerph13010086

**Published:** 2015-12-29

**Authors:** Tao Zhang, Jian Du, Xiaoyan Yin, Fuzhong Xue, Yanxun Liu, Runzi Li, Cheng Luo, Liang Li, Xiujun Li

**Affiliations:** 1 Department of Biostatistics, School of Public Health, Shandong University, Jinan 250012, China; tao_zhang@live.com (T.Z.); xuefzh@sdu.edu.cn (F.X.); liu-yx@sdu.edu.cn (Y.L.); runzi_li@hotmail.com (R.L.); luomadcheng@vip.163.com (C.L.); 2 Clinical Center Office, Beijing Tuberculosis and Thoracic Tumor Research Institute, Beijing 101149, China; jdu-sdu@163.com (J.D.); liliang@tb123.org (L.L.); 3 Beijing Chest Hospital, Capital Medical University, Beijing 101149, China; 4 National Heart Lung and Blood Institute’s and Boston University’s Framingham Heart Study, Framingham, MA 01702-5827, USA; xyin@bu.edu

**Keywords:** adverse events, tuberculosis epidemiology, risk factors, China

## Abstract

This study aimed to estimate the adverse events (AE) rate during anti-tuberculosis treatment and to explore AE-related risk factors. New and previously treated smear-positive tuberculosis (TB) cases were enrolled from eight regions in China between April 2009 and October 2010. The AE rate was estimated, and AE risk factors during anti-TB treatment were assessed using Cox proportional models. Among 2091 Chinese subjects with anti-TB treatment, 462 (22.1%, 95% confidence interval (CI), 20.3–23.9) patients developed AE, with liver injury and gastrointestinal reactions constituting the most common AE. Specifically, 9.8% (95% CI, 8.5–11.1) and 6.3% (95% CI, 5.3–7.4) developed liver injuries and gastrointestinal reactions, respectively. We found that AE rate differed by regions, TB knowledge score, symptoms score and smoking status. Liver injuries were associated with age, sex and smoking status; gastrointestinal reactions were associated with education level and symptom score. Improving patients’ knowledge on TB could reduce AE rate.

## 1. Introduction

Tuberculosis (TB) remains a major health problem around the world, especially in China. According to the World Health Organization (WHO), there were an estimated 8.5 to 9.2 million incident TB cases and 1.2 to 1.5 million TB-related deaths in 2010 [1]. India and China had the world’s largest TB epidemic, together accounting for 40% of the world’s notified cases in 2010. China had approximately 1.0 million TB cases in 2013, and the prevalence of pulmonary TB was 66/100,000 according to the fifth national survey [2,3].

Patients diagnosed with pulmonary TB should receive anti-TB treatment. During treatment, TB patients may suffer from adverse events (AE), such as gastritis, hepatitis and even fatal liver injuries [4,5]. AE is associated with increased risk for prolonged course of treatment or even unsuccessful treatment outcomes [6,7]. The reported incidences of AE range from 17% to 35% [8,9,10,11]. Despite the severity of AE, the number of studies exploring AE risk factors during anti-TB treatment has been limited. Only 245 papers were found in PubMed [12] until March 2015 when “’Antitubercular Agents/adverse effects’ (MeSH) AND ‘Risk Factors’ (MeSH)” was used as search term, among which merely 11 were from China. Most of these studies only analyzed one or two specific AE, such as hepatotoxicity, and were also limited by small sample sizes and local study areas [4,13,14,15]. Moreover, very few studies explored the risk factors of AE by combining data of socio-demographic characteristics, clinical characteristics and laboratory data.

With the current rapid economic growth and medical service development in China, there has been a significant improvement for TB prevention, control and management. The incidence and risk factors of AE may have changed. Different adverse events may have common risk factors, and it is necessary to identify them. Therefore, we conducted this study among Chinese TB patients with the aim of estimating the AE rate and identifying its risk factors.

## 2. Materials and Methods

### 2.1. Sampling Method

This study was conducted and supervised by Beijing Chest Hospital. Newly registered TB cases and previously treated smear-positive TB cases were enrolled consecutively in this study between April 2009 and October 2010.

According to the location, implementation ability and epidemic pattern of pulmonary TB, 48 sites in eight regions were chosen and were divided into three regions, including east, center and west region. Specifically, the east included Shanghai and Guangzhou; the center included Tianjin, Henan, Hebei and Chongqing; and the west included Guangxi and Yunnan. The east is more economically developed than the center and the west. This study was approved by the Ethics Committee of Beijing Chest Hospital, and conducted in accordance with the principles of the Declaration of Helsinki of the World Medical Association. Written informed consent was obtained from each participant.

### 2.2. Data Collection

The new TB cases were interviewed at baseline (the first day when one received treatment) and the 1st, 2nd, 5th and 6th month during the treatment, and the previously treated patients were interviewed at baseline and the 1st, 2nd, 5th and 8th month during the treatment. Samples of sputum were collected for smear and culture at baseline, and blood samples were collected for routine blood examinations and liver/kidney function tests at baseline and each interview. Socio-demographic characteristics and treatment history of the enrolled patients were recorded by face-to-face interview at baseline. Drug susceptibility testing (DST) of mycobacterium TB was carried out among the patients with positive sputum smears. The adverse events occurring during anti-TB treatment were self-reported by the patients and then checked by medical specialists at each interview. All information was first recorded in the case report forms when a patient was interviewed, and then inputted into the online electronic information system by well-trained doctors. The electronic information system was constructed according to the case report form, and the same system was used at all sites.

Patients’ symptoms were evaluated by the doctors according to a symptoms score at baseline. A lower score indicated fewer symptoms. We also designed specific questions to assess the patients’ knowledge on TB using a specifically designed score system. A higher score indicated more knowledge on TB (see Supplementary Material 1, which contains questions for evaluation of tuberculosis knowledge, and the evaluation standard of tuberculosis knowledge and various symptoms).

Current smokers were defined as those who had smoked at least 1 cigarette a day for at least 1 year and still smoked during the past year. Ex-smokers were defined as those who had smoked at least 1 cigarette a day for at least 1 year but had stopped smoking for at least 1 year before the study. Never smokers were defined as those who were neither current smokers nor ex-smokers. Similarly, current drinkers were defined as those who had drunk on 4 or more days per week for at least 1 year and still drank during the past year. Ex-drinkers referred to those who drank on 4 or more days per week for at least one year but had stopped drinking before the study. Never drinkers were defined as those who were neither current drinkers nor ex-drinkers [16]. In this study, all of the adverse events were classified into 12 categories as following:

(I) Liver injury, including elevated transaminase and jaundice.
(II) Gastrointestinal reactions, including nausea, vomiting, loss of appetite and diarrhea.
(III) Renal impairment, including decline of renal function, positive urine protein and renal failure.
(IV) Blood system damage, including anemia, leukopenia, thrombocytopenia, and pancytopenia.
(V) Auditory nerve damage, including tinnitus, dizziness and hearing loss.
(VI) Optic nerve damage, including blurred vision, constriction of visual field, and color vision disorder.
(VII) Drug allergy, including pruritus, rash, exfoliative dermatitis, and anaphylactic shock.
(VIII) Neuropsychiatric symptoms, including sleep disorders, dizziness, headache, irritability, delirium and epilepsy.
(IX) Joints or muscle pain, including arthralgia and Achilles pain.
(X) Electrolyte abnormalities.
(XI) Thyroid dysfunction.
(XII) Other adverse events.

### 2.3. Bacteriological Examinations

The bacteriological examinations were implemented based on “China TB control program: handbook of sputum smear quality assurance in microscopy room.” Löwenstein-Jensen medium was used for sputum culture, and proportion method was used for DST (see Supplementary Material 2, which contains more details about bacteriological examinations). Patients with non-tuberculous mycobacteria (NTM) infection were excluded from the study.

### 2.4. Anti-Tuberculosis Therapy

All of the enrolled subjects were treated with a daily regimen or intermittent short-course regimen and recommended doses according to *Guidelines for Implementing the National Tuberculosis Control Program in China* (2008) [17] (see Supplementary Material 2, which contains more details about anti-tuberculosis therapy). No additional anti-TB drugs except the regimens mentioned in the Supplementary Material 2 were used.

### 2.5. Statistical Analysis

We studied three outcomes in this study: overall AE and the two separate common AEs, liver injuries and gastrointestinal reactions. The three outcomes were defined as follows:

Scenario 1: The outcome was overall AE which was defined to be true if an AE of any category listed above in *2.2 Data Collection* occurred;

Scenario 2: The outcome was liver injury which was defined to be true if any category I AE occurred;

Scenario 3: The outcome was gastrointestinal reaction which was defined to be true if any category II AE occurred.

For each patient the time to event was defined as the time from baseline to his/her earliest outcome in each scenario. For patients without the outcome of interest, the follow-up was censored at the earliest of last treatment, loss of track or study end. Both AE rate and incidence density, along with their 95% confidence intervals (CI), were calculated to describe the frequency of AE occurring among TB patients [18]. AE rate was calculated as the proportion of TB cases with AE outcome among total subjects, and incidence density was calculated as the number of TB cases with AE outcome per 100 person-months of follow-up. Each of the potential risk factors was evaluated for its association with AE using a Cox proportion hazard model. Then stepwise selection was used in multivariable adjusted Cox models to identify a panel of risk factors of AE. The factors with *p*-value < 0.2 in single risk factor analyses were included in multivariable analysis as candidate risk factors. The statistical analyses were performed using SAS software (Statistical Analysis System, version 9.4; SAS, Cary, NC, USA). We used two-sided *p*-value < 0.05 as criterion for significance.

## 3. Results

In total we recruited 2142 patients, of whom 2091 were included in this analysis. The other 51 patients were excluded because of NTM, negative sputum smear or voluntary withdrawal. About 40 patients were recruited at each site. The average follow-up time was 7.2 (±2.2) months.

### 3.1. Socio-Demographics Characteristics

Of 2091 patients, 1511 (72.3%) were male, and 1695 (81.1%) were Han Chinese. The average age was 43.1 (±17.3) years, with 65 patients (3.1%) younger than 18 and 393 (18.8%) older than 60. A total of 1355 (64.8%) patients had no more than a secondary school diploma as their highest level of education. There were 955 (46.5%) never smokers, 741 (36.1%) ex-smokers and 357 (17.4%) current smokers. There were 1133 (55.2%) never drinkers, 747 (36.4%) ex-drinkers and 171 (8.3%) current drinkers (Table 1). As for spatial distribution, the east region had 658 (31.5%) of total subjects, the center had 824 (39.4%), and the west had 609 (29.1%).

**Table 1:** Baseline characteristics of the patients (*n* = 2091) and results of single risk factor analysis.

Characteristics	Patients without AE	Patients with AE	Overall	*p*
	No. (%)	No. (%)	No. (%)	
**Age, years**	1625 (100)	460 (100)	2085 (100)	
Mean ± SD	42.3 ± 17.3	45.6 ± 17.3	43.1 ± 17.3	
≤18	52 (3.2)	13 (2.8)	65 (3.1)	**0.05**
19–39	708 (43.6)	167 (36.3)	875 (42.0)	† §
40–59	572 (35.2)	180 (39.1)	752 (36.1)	
≥60	293 (18.0)	100 (21.7)	393 (18.8)	
**Sex**	1629 (100)	462 (100)	2091 (100)	
Male	1176 (72.2)	335 (72.5)	1511 (72.3)	0.74
Female	453 (27.8)	127 (27.5)	580 (27.7)	†¶
**Treatment history**	1629 (100)	462 (100)	2091 (100)	
New cases	1379 (84.7)	398 (86.1)	1777 (85.0)	0.29
Previously treated	250 (15.3)	64 (13.9)	314 (15.0)	†
**Ethnic groups**	1629 (100)	462 (100)	2091 (100)	
Han	1331 (81.7)	364 (78.8)	1695 (81.1)	0.13
Minority	298 (18.3)	98 (21.2)	396 (18.9)	§
**Level of education**	1629 (100)	462 (100)	2091 (100)	
Illiteracy or primary school	549 (33.7)	187 (40.5)	736 (35.2)	**<0.01**
Middle school or higher	1080 (66.3)	275 (59.5)	1355 (64.8)	† §
**Region**	1629 (100)	462 (100)	2091 (100)	
East	514 (31.6)	144 (31.2)	658 (31.5)	**<0.01**
Center	681 (41.8)	143 (31.0)	824 (39.4)	† §
West	434 (26.6)	175 (37.9)	609 (29.1)	
**Smoking status**	1594 (100)	459 (100)	2053 (100)	
Never smokers	760 (47.7)	195 (42.5)	955 (46.5)	**0.02**
Ex-smokers	573 (36.0)	168 (36.6)	741 (36.1)	†¶
Current smokers	261 (16.4)	96 (20.9)	357 (17.4)	
**Drinking status**	1593 (100)	458 (100)	2051 (100)	
Never drinkers	894 (56.1)	239 (52.2)	1133 (55.2)	0.24
Ex-drinkers	569 (35.7)	178 (38.9)	747 (36.4)	†
Current drinkers	130 (8.2)	41 (9.0)	171 (8.3)	
**DOT distance, kilometer**	1588 (100)	459 (100)	2047 (100)	
<1	547 (34.5)	118 (25.7)	665 (32.5)	**<0.01**
1–5	566 (35.6)	189 (41.2)	755 (36.9)	†
6–10	276 (17.4)	79 (17.2)	355 (17.3)	
>10	199 (12.5)	73 (15.9)	272 (13.3)	
**DOT supervisor**	1591 (100)	452 (100)	2043 (100)	
Doctors	889 (55.9)	251 (55.5)	1140 (55.8)	0.91
Others	679 (42.7)	194 (42.9)	873 (42.7)	
Nobody	23 (1.4)	7 (1.6)	30 (1.5)	
**TB knowledge**	1629 (100)	462 (100)	2091 (100)	
Mean ± SD	11.3 ± 3.6	10.4 ± 3.8	11.1 ± 3.6	
≤5	134 (8.2)	63 (13.6)	197 (9.4)	**<0.01**
6–10	458 (28.1)	146 (31.6)	604 (28.9)	†§
>10	1037 (63.7)	253 (54.8)	1290 (61.7)	
**Symptom score**	1629 (100)	462 (100)	2091 (100)	
Mean ± SD	4.8 ± 2.9	5.7 ± 3.5	5.0 ± 3.1	
≤5	1082 (66.4)	247 (53.5)	1329 (63.6)	**<0.01**
6–10	476 (29.2)	168 (36.4)	644 (30.8)	§
>10	71 (4.4)	47 (10.2)	118 (5.6)	
DST	1041 (100)	333 (100)	1374 (100)	
Sensitive	691 (66.4)	232 (69.7)	923 (67.2)	0.17
Resistant	350 (33.6)	101 (30.3)	451 (32.8)	

Notes: * Exact *p* values were shown in Supplementary Material 3. † means *p* < 0.05 in multivariable analysis for liver injury; ‡ means 0.05 < *p* ≤ 0.2 in multivariable analysis for liver injury; § means *p* < 0.05 in in multivariable analysis for gastrointestinal reactions; ¶ means 0.05 < *p* ≤ 0.2 in multivariable analysis for gastrointestinal reactions. Definition of abbreviations: TB = tuberculosis; DOT = directly observed therapy; DST = Drug susceptibility testing.

### 3.2. Clinical Characteristics

A total of 1777 (85.0%) subjects were new TB cases. The average symptom score was 5.0 (±3.1). There were 1329 patients (63.6%) with a symptom score ≤5, and 118 patients (5.6%) had a score >10. For knowledge score, the average level was 11.1 (±3.6). There were 197 (9.4%) subjects with a knowledge score ≤5, whereas 1290 (61.7%) had a score >10.

In this study, 1420 (69.4%) patients had a distance of no more than 5 kilometers between the place of directly observed therapy (DOT) and their residences. During directly observed treatment, there were 1140 (55.8%) patients supervised by doctors, while 30 (1.5%) were not supervised.

DST results were available among 1367 patients, of whom 923 (67.2%) were pansensitive TB, and 451 (32.8%) were drug-resistance.

### 3.3. Adverse Events

During anti-TB treatment, 462 patients suffered from AE. The AE rate was 22.1% (95% CI, 20.3–23.9), and the incidence density was 3.1 per 100 person-months (95% CI, 2.8–3.3) among the TB cases.

**Figure 1:**
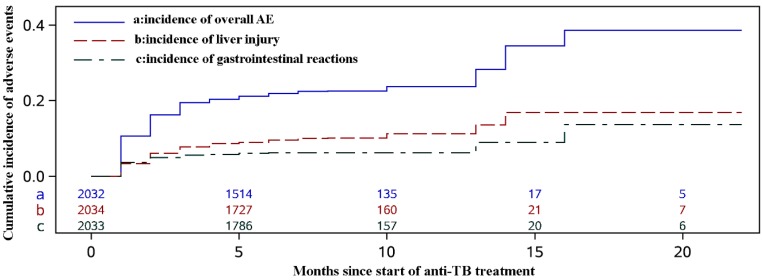
Cumulative probability of adverse events (AE) since start of anti-TB treatment.

Among the patients with AE, the average time between onset of anti-TB treatment and first-time adverse events occurrence was 2.2 (±2.0) months. As shown in Table 2, the most common adverse event was liver injury, followed by gastrointestinal reactions. The rate of liver injury was 9.8% (95% CI, 8.5–11.1), and the incidence density was 1.4 per 100 person-months (95% CI, 1.2–1.6). The rate of gastrointestinal reactions was 6.3% (95% CI, 5.3–7.4), and the incidence density was 0.9 per 100 person-months (95% CI, 0.7–1.0). The two events accounted for 60.7% of the total adverse effects. No thyroid dysfunction was reported throughout the follow-up. The cumulative probability curves are shown in Figure 1.

**Table 2:** The frequency and proportion of adverse events during anti-tuberculosis treatment.

AE Groups	No.	%
Liver injury *	205	36.94
Gastrointestinal reactions *	132	23.78
Renal impairment	28	5.05
Blood system damage	18	3.24
Auditory nerve damage	4	0.72
Optic nerve damage	15	2.70
Drug allergy	69	12.43
Neuropsychiatric symptoms	37	6.67
Joints/Muscle pain	35	6.31
Electrolyte abnormalities	1	0.18
Thyroid dysfunction	0	0
Other adverse events	11	1.98

Note: * There were 11 patients overlapping between these two groups, and the liver function test values of patients with liver injuries and patients with gastrointestinal reactions were described in Supplementary Material 4.

### 3.4. Risk Factors for Adverse Events during Anti-TB Treatment

Seven factors were individually associated with AE, including age, level of education, region, smoking, DOT distance, TB knowledge and symptom score, as shown in Table 1. In multivariable analysis, four factors stayed in the final model. TB knowledge (hazard ratio (HR), 0.69; 95% CI, 0.60–0.81) was a protective factor, whereas the other three factors were associated with increased risk of overall AE, including region (west *vs.* center, HR, 1.82; 95% CI, 1.35–2.45), smoking (current *vs.* never smokers, HR, 1.64; 95% CI, 1.23–2.18) and symptom score (HR, 1.05; 95% CI, 1.02–1.09).

Risk factors for liver injury included region (east *vs.* center, HR, 1.91, 95% CI, 1.35–2.71; west *vs.* center, HR, 1.54, 95% CI, 1.06–2.23), age (≥60 *vs.* 19–39, HR, 0.50, 95% CI, 0.32–0.80), sex (male *vs.* female, HR, 1.66, 95% CI, 1.08–2.54), smoking (current *vs.* never smokers, HR, 1.71, 95% CI, 1.12–2.59) and TB knowledge (HR, 0.79, 95% CI, 0.65–0.97), whereas risk factors for gastrointestinal reactions included region (east *vs.* center, HR, 0.34, 95% CI, 0.19–0.62), level of education (HR, 0.44, 95% CI, 0.30–0.65), symptom score (HR, 1.09, 95% CI, 1.03–1.14) and TB knowledge (HR, 0.66, 95% CI, 0.52–0.84) (Table 3).

**Table 3:** The adjusted hazard ratios (HR) of risk factors for adverse events (AE) in multivariable analyses.

Characteristics	Overall AE			Liver Injury			Gastrointestinal Reactions		
	HR	95% CI	*p*	HR	95% CI	*p*	HR	95% CI	*p*
Region									
East	1.33	0.97–1.83	0.07	1.91	1.35–2.71	< 0.01	0.34	0.19–0.62	<0.01
West	1.82	1.35–2.45	< 0.01	1.54	1.06–2.23	0.02	0.77	0.52–1.14	0.19
Center	ref			ref			ref		
Age, years									
≤18	–	–	–	1.50	0.75–3.00	0.25	–	–	–
40–59	–	–	–	0.79	0.57–1.08	0.14	–	–	–
≥60	–	–	–	0.50	0.32–0.80	< 0.01	–	–	–
19–39				ref					
Sex (Male)	–	–		1.66	1.08–2.54	0.02	–	–	–
Education level	–	–		–	–	–	0.44	0.30–0.65	<0.01
Smoking status									
ex-smokers	1.20	0.94–1.55	0.15	1.27	0.87–1.85	0.21	–	–	–
current smokers	1.64	1.23–2.18	< 0.01	1.71	1.12–2.59	0.01	–	–	–
never smokers	ref			ref					
Symptom score	1.05	1.02–1.09	0.01	–	–	–	1.09	1.03–1.14	<0.01
TB Knowledge	0.69	0.60–0.81	< 0.01	0.79	0.65–0.97	0.02	0.66	0.52–0.84	<0.01

## 4. Discussion

Our study thoroughly assessed AE outcome rates and risk factors during anti-TB treatment among Chinese TB patients. The rate of overall AE was 22.1%, among which liver injury was the most common AE, followed by gastrointestinal reactions.

A few previous studies analyzed adverse events during anti-TB treatment in China, but the subjects in these studies were recruited from either a single or regional location and sample sizes were small [5,6,9]. Moreover, to date there are no published reports on current nationwide incidence of AE during anti-TB treatment. This study was also conducted on a much larger scale than previous studies: more than 2000 TB patients from broad areas with distinct economic levels in China.

The AE rate estimated by our study was comparable to those from previous studies [10,11,19]. Liver injury was the most studied AE in anti-TB treatment [14,20,21]. In Taiwan, the incidence of liver injury was 12.0% among TB patients [15]. In mainland China, Xia *et al.* reported an incidence of 11.9% by reviewing the reports published during 1996–2005 [11]. In our study 9.8% of total TB cases developed liver injury. The reduction may be due to the improvement of health care and medical treatment level. Although it was reported that rifampicin could cause hypothyroidism, no case with thyroid dysfunction was found in our study [22].

The difference in AE rates among various regions suggested unbalanced spatial distribution of AE incidence. Previous studies showed that the eastern China had the lowest prevalence of TB, whereas the west had the highest prevalence [23]. However, we did not find the same incidence pattern for all of the AE outcomes. For instance, we found that the central region had lower incidence of liver injuries compared with the east and west regions, whereas the east region had the lowest rate of gastrointestinal reactions. This finding may be attributable to the variability of available medical resources and lifestyles in different regions. Medical policymakers should pay attention to prevalent liver injuries resulting from anti-TB treatment in the east and the west regions, and to common gastrointestinal reactions in the central region.

Age is an important risk factor for adverse events, likely due to decreased ability to metabolize drugs [24]. However, we did not detect an increased risk for AE among the aged during anti-TB treatment. Compared with the 19–39 year–old group, age over 60 years was a protective factor for liver injury. This might be attributed to the confounding effect of hepatoprotective drugs usage, as some doctors prescribed hepatoprotective drugs in advance to prevent liver injury. Unfortunately this explanation cannot be verified because we lacked information about other drugs used. In addition, the unhealthy lifestyle, such as irregular resting schedule in young people, could increase the risk of liver injury [25].

Patients’ education level also plays an important role in the occurrence of adverse events during anti-TB treatment. We found that higher level of education (middle school and higher) was a protective factor for gastrointestinal reactions. The finding was consistent with the study of Przybylski *et al.* [26], indicating that the knowledge-attitude-behavior model is a probable explanation for the effects of education on AE. Hofman *et al.* found cancer patients with college education suspected themselves of suffering from more treatment–related side effects than patients with high school education or lower [27]. Similarly, it is possible that TB patients with different education levels have different expectation for adverse events, and thus have different self-interventions that may have affected the occurrence of AE.

China is a multi-ethnic country, with the Han Chinese as the majority. In this study, the AE incidence for Han Chinese was slightly higher than that for ethnic minorities, but the difference was not significant.

We found that smoking was significantly associated with increased risk of AE occurrence during anti-TB treatment, especially liver injury. *In vivo* murine studies have found that mice exposed to cigarette smoke exhibit greater bacterial burden, fewer host-protective macrophages and dendritic cells and fewer interferon-gamma (IFN-γ) positive T cells which also protect the host in lungs [28,29,30,31]. These findings suggests that smoking may impair anti-TB immunity. It should be pointed out that no significant difference in AE risk was found between ex-smokers and never smokers.

We found that TB patients with higher symptom scores showed increased risk for overall adverse events and gastrointestinal reactions. Every 1 unit increase in symptom score was associated with 1.09 fold increased risk for gastrointestinal reactions. Higher symptom scores usually mean sicker patients. It is possible to be attributed to weak immunity, but this supposition requires further evidence from laboratory and clinical epidemiological studies. Also, we did not find a significant association between symptom score and liver injury.

Health education for TB patients before treatment had been proposed as an important step of DOT implementation in China [32]. In the latest edition of *National Guideline on Tuberculosis Control Program (2011–2015)*, China has clearly prioritized enhancing health education on TB as a national goal. In this study, we found that more knowledge on TB was a protective factor for AE among TB patients. In addition, when the effect of education level was adjusted, the conclusion was the same. Dick *et al.* found that health education could increase patients’ adherence to treatment, which is important to the outcome of anti-TB treatment [33,34]. Thus, health education among TB patients not only benefits TB patients by improving treatment success, but it also protects TB patients from AE during anti-TB treatment.

Female sex was reported as an independent predictor for anti-TB drug-induced AE [15,35]. However, this has not been consistently verified by other studies, especially those involving TB patients from third world countries [36,37,38]. We did not replicate this association either; on the contrary, males had higher risk for liver injury in our study. Other studies focusing on Chinese TB patients also reported that males had higher AE incidence than females, though the difference was not significant after adjustment [35,37]. In China, men usually have more social activities and life burden than women and thus are more likely to be sub-healthy, providing an explanation for why we observed more AE in men than in women.

Though the AE incidence was higher in pansensitive TB patients than that in drug-resistance TB patients, the difference was not statistically significant. According to Yee *et al.*, drug resistance was only associated with AE to isoniazid [39]. However, the difference is difficult to identify when the adverse events were induced by a mix of drugs in this study.

In this study, all new cases adopt the 6-month regimen, and all previously treated patients were treated with the 8-month regimen. Single risk factor analysis showed that treatment history was not associated with AE, which indicated that the treatment regimen was not likely a risk factor for AE.

There are several limitations in our study. As noted above, the patients in this study were treated with a mix of several drugs. Thus, it is difficult to analyze the relationships between the AE and specific drugs separately. Other studies found that some diseases, such as diabetes mellitus, hepatitis B virus and human immunodeficiency virus (HIV) infection could affect the occurrence of AE, but we lacked information about these diseases for our selected patients [40,41,42]. Some patients had courses of treatment longer than the prescription because of various reasons. However, the adherence rate to medication was not assessed due to the lack of an evaluation variable. In addition, the missing values in DST results were substantial (proportion of missing values was 34%) and we decided not to impute missing values.

## 5. Conclusions

In summary, the overall adverse events rate in Chinese TB patients was 22.1% (95% CI, 20.3–23.9), 9.8% (95% CI, 8.5–11.1) for liver injury and 6.3% (95% CI, 5.3–7.4) for gastrointestinal reactions. Unbalanced spatial distribution of AE during anti-TB treatment course was significant in China. TB knowledge acts as a protective factor for AE. These findings suggest that Chinese TB patients could benefit from the government’s further work on improving medical resource allocation and patients’ knowledge on TB on a national scale.

## Supplementary Files

Supplementary File 1
