# Supplementary material for: Adverse Events in Treating Smear-Positive Tuberculosis Patients in China
Source: Int J Environ Res Public Health. 2015 Dec 29;13(1):86. doi: 10.3390/ijerph13010086 (PMC4730477; doi:10.3390/ijerph13010086)
Supplement: Supplementary File 1 [file ijerph-13-00086-s001.pdf]

Supplementary Materials: Adverse Events in Treating Smear-Positive Tuberculosis Patients in China

Tao Zhang, Jian Du , Xiaoyan Yin, Fuzhong Xue, Yanxun Liu, Runzi Li, Cheng Luo, Liang Li and Xiujun Li

Supplementary Material 1

Questions for Evaluation of Tuberculosis Knowledge

Q1. Do you know that tuberculosis is chronic respiratory infectious disease that results in serious health problem?

(1) No, I don't know (never heard it).

(2) Yes, I know.

Q2. How long does a patient cough last, then he is suspected to get tuberculosis?

(1) <2 weeks.

(2) ≥2 weeks.

(3) >1 month.

(4) I don't know.

Q3. Which place is the best choice for a suspected tuberculosis patient?

(1) I don't know.

(2) Private clinics.

(3) Village health station/community health station.

(4) Town health center/community health center.

(5) County/city/provincial general hospital.

(6) Centers for tuberculosis control and prevention/centers for disease control and prevention/tuberculosis hospital.

Q4. Is it free to diagnose and treat tuberculosis in county centers for tuberculosis control and prevention?

(1) Yes, it is.

(2) No, it isn't.

(3) I don't know about this.

Q5. Is tuberculosis curable when a tuberculosis patient adheres to regular treatment?

(1) I don't know.

(2) No, it isn't.

(3) Most cases is curable.

(4) Yes, it is.

Table S1. Scores for different options in the question above.

	Option 1	Option 2	Option 3	Option 4	Option 5	Option 6
Q1	0	3				
Q2	0	3	1	0		
Q3	0	0	1	1	1	3
Q4	3	0	0			
Q5	0	0	1	3		

Table S2. Evaluation standard of various symptoms.

**Symptoms**	**Score**			
	**0**	**1**	**2**	**3**
cough	none	Intermittent cough during the day, but it doesn't disturb routine work and life		Frequent cough day and night, and it affects work and sleep
sputum	≤20 mL per day	20–50 mL per day	50–100 mL per day	>100 mL per day
hemoptysis	none	<50mL per time	50–200 mL per time	>200 mL per time
chest pain	none	dull pain, 2 to 3 times per day	relative intense pain, over 3 times per day, and it affects life	persistent severe pain, and it affects sleep
dyspnea	none	feel a little difficulty in breathing, worse after physical activities, but don't need oxygen therapy	affects rest, and get better after oxygen therapy	the symptoms at 2 with cyanosis/pale, heart failure/coma, and oxygen therapy can't give a relief
fatigue	none	mild	moderate	severe
weight reduction	none	mild	moderate	severe
fever	none	<38 °C	38 °C –40 °C	>40 °C

Supplementary Material 2

Details for Bacteriologic Examinations

Before initiation of the treatment, every eligible patient was collected spot sputum, morning sputum and night sputum. Sputum smears were stained with Ziehl-Neelsen reagents. Sputum culture used Löwenstein-Jensen medium. If there were no bacteria growing until the 8th week after inoculation, the culture was recorded as negative.

DST was performed by using the proportion method, with the following concentrations for the six anti-TB drugs: 0.2 μg/mL for isoniazid (INH, H), 40 μg/mL for rifampicin (RFP, R), 2.0 μg/mL for ethambutol (EMB, E), 4.0 μg/mL for streptomycin (SM, S), 30 μg/mL for kanamycin (KM), and 2 μg/mL for of ofloxacin (OFX). If a patient was resistant to anyone of these drugs, he/she would be recorded as drug-resistance. At the same time, paranitrobenzoic acid (PNB) was used to differentiate between Mycobacterium TB and non-tuberculous mycobacteria (NTM).

Regimens and Doses for Tuberculosis Used in This Study

(1) Regimens Choice:

New cases: $2H_3R_3E_3Z_3/4H_3R_3$ or daily regimen

Previously treated cases: $2S_3H_3R_3E_3Z_3/6H_3R_3E_3$ or daily regimen

(2) Doses:

Table S3. Doses in different regimens.

Drugs	Daily Regimen			Intermittent Short-Course Regimen	
	Adults (g)		Children	Adults (g)	
	<50 kg	≥50 kg	(mg/kg)	<50 kg	≥50 kg
Isoniazid	0.3	0.3	10–15	0.6	0.6
Streptomycin	0.75	0.75	20–30	0.75	0.75
Rifampicin	0.45	0.6	10–20	0.6	0.6
Ethambutol	0.75	1.0	-	1.0	1.25
Pyrazinamide	1.5	1.5	30–40	1.5	2.0

Supplementary Material 3

Table S4. Single risk factor analysis *p*-values.

	p Value		
	**Overall AE (n = 462)**	**Liver Injuries (n = 205)**	**Gastrointestinal Reactions (n = 132)**
Region	<0.01	<0.01	<0.01
Education level	<0.01	0.04	<0.01
DOT supervisor	0.91	0.23	0.93
DST	0.18	0.55	0.74
Drinking	0.24	0.02	0.66
Treatment history	0.29	0.02	0.60
DOT distance	0.003	0.01	0.50
Sex	0.74	0.001	0.19
Smoking	0.02	0.003	0.11
Ethnic group	0.13	0.98	0.02
TB knowledge	<0.01	0.04	<0.01
Age	0.05	0.03	<0.01
Symptom score	<0.01	0.96	<0.01

Supplementary Material 4

Table S5. The median values with percentiles (P25–P75) of liver function test values among patients with liver injuries and patients with gastrointestinal reactions *.

Indexes	Median (P25–P75) among Patients with Liver Injuries (n = 205)	Median (P25–P75) among Patients with Gastrointestinal Reactions (n = 132)
Aspartate transaminase, U/L	41.0 (30.0–60.8)	30.0 (24.0–36.5)
Alanine transaminase, U/L	45.0 (26.0–79.0)	27.0 (18.0–31.0)
Total bilirubin, μmol/L	19.3 (14.7–24.9)	13.0 (10.6–16.7)
Indirect bilirubin, μmol/L	12.7 (8.3–17.0)	8.0 (5.5–11.8)

Note: * The liver function test value used within a patients was the highest one of the test values at 5 interviews.

© 2016 by the authors; licensee MDPI, Basel, Switzerland. This article is an open access article distributed under the terms and conditions of the Creative Commons by Attribution (CC-BY) license (http://creativecommons.org/licenses/by/4.0/).
